# Kefir metabolites in a fly model for Alzheimer’s disease

**DOI:** 10.1038/s41598-021-90749-8

**Published:** 2021-05-27

**Authors:** Letícia Leandro Batista, Serena Mares Malta, Heitor Cappato Guerra Silva, Luiza Diniz Ferreira Borges, Lays Oliveira Rocha, Jéssica Regina da Silva, Tamiris Sabrina Rodrigues, Gabriela Venturini, Kallyandra Padilha, Alexandre da Costa Pereira, Foued Salmen Espindola, Carlos Ueira-Vieira

**Affiliations:** 1 Institute of Biotechnology, Universidade Federal de Uberlândia, Uberlândia, Brazil; 2 Department of Genetics, Harvard Medical School, Boston, MA USA; 3 Laboratory of Genetics and Molecular Cardiology, Heart Institute, University of São Paulo Medical School, São Paulo, SP Brazil

**Keywords:** Diseases of the nervous system, Microbial communities

## Abstract

Alzheimer’s Disease (AD) is the most common cause of dementia among elderly individuals worldwide, leading to a strong motor-cognitive decline and consequent emotional distress and codependence. It is traditionally characterized by amyloidogenic pathway formation of senile plaques, and recent studies indicate that dysbiosis is also an important factor in AD’s pathology. To overcome dysbiosis, probiotics—as kefir—have shown to be a great therapeutic alternative for Alzheimer’s disease. In this present work, we explored kefir as a probiotic and a metabolite source as a modulator of microbiome and amyloidogenic pathway, using a *Drosophila melanogaster* model for AD (AD-like flies)*.* Kefir microbiota composition was determined through 16S rRNA sequencing, and the metabolome of each fraction (hexane, dichloromethane, ethyl acetate, and n-butanol) was investigated. After treatment, flies had their survival, climbing ability, and vacuolar lesions accessed. Kefir and fraction treated flies improved their climbing ability survival rate and neurodegeneration index. In conclusion, we show that kefir *in natura*, as well as its fractions may be promising therapeutic source against AD, modulating amyloidogenic related pathways.

## Introduction

Alzheimer’s Disease (AD) is the most common cause of dementia among elderly individuals worldwide, leading to a strong cognitive decline and consequent emotional distress and codependence^1^. Its pathophysiology is multifactorial, but traditionally characterized by senile plaques production and deposit through the amyloidogenic pathway. In this pathway, the amyloid precursor protein (APP) is cleaved by the β-secretase enzyme, generating an Aβ peptide of 40 and 42 amino acids^2^, which oligomerizes and fibrilizes, causing senile plaques and leading to synapse degeneration^3^.

Recent studies indicate that dysbiosis—characterized by host gut-microbiome disbalance—plays a big role in AD’s pathology^4–6^. These alterations may contribute to neuronal damage by inhibiting pathways related to Aβ clearance, or directly by improving this peptides production or accumulation^7–9^.

To overcome dysbiosis, probiotics have shown to be a great therapeutic alternative for Alzheimer’s Disease^10^. Within this approach, kefir—natural probiotic drink constituted by symbiotic bacteria and yeasts—has been used^11,12^. It uses milk as a substrate, producing metabolic molecules with health-improving effects, as antioxidant, and anti-inflammatory properties^13–18^. Kefir administration has shown to be able to attenuate AD effect both in rats, through inflammatory process modulations^19,20^, and in AD patients, improved cognitive function and lowering both oxidative stress levels and red cells damage^21^.

As *Drosophila melanogaster* shares a similar yet simpler central nervous system in relation to mammals, its use in investigating neurodegenerative diseases has been incredibly valuable^22–25^. Plus, it has been suggested to be an interesting model for exploring gut-brain-axis interactions within these diseases^26,27^. Studies have shown that probiotic treatment attenuates AD effects^27^, but no study has investigated probiotics metabolites on AD model.

No previous studies have explored the effects of a peptide and cell-free fraction of kefir, compared its effects to the *in natura* version, or evaluated its metabolome. This way, in this study, we explored kefir effects—and from its metabolites—in *D. melanogaster* expressing human BACE and APP.

## Results

### Kefir characterization

Since kefir grains bacterial community may vary depending on its source and preparation, to obtain a fingerprint of our sample, next-generation sequencing (NGS) was used to analyze the prokaryotic 16S ribosomal DNA gene (v4 region). A total of 180,388 paired-end V4-16S raw reads were obtained from kefir grains with its fermentation product. After pre-processing, 143,961 reads were kept, with an average length of 252 bp. Processed sequences were clustered with representative sequences, and a 97% sequence identity cut-off was used. From this, five Operational Taxonomic Units (OTUs) were generated and sequences were BLAST against the NCBI nucleotide collection for taxa verification (Table 1).

**Table 1:** 16S-v4 read abundance and taxonomic units.

Taxonomy	Read abundance (total)	Read abundance (%)
Bacteria; Firmicutes; Bacilli; Lactobacillales; Lactobacillaceae; *Lactobacillus; Lactobacillus kefiranofaciens*	31,980	21.96
Bacteria; Firmicutes; Bacilli; Lactobacillales; Lactobacillaceae; *Lactobacillus; Lactobacillus kefiri*	294	0.20
Bacteria; Proteobacteria; Alphaproteobacteria;Rhodospirillales; Acetobacteraceae; *Acetobacter; Acetobacter fabarum*	261	0.17
Bacteria; Firmicutes; Bacilli; Lactobacillales;Streptococcaceae; *Lactococcus; Lactococcus lactis*	7	0.004
Bacteria; Proteobacteria; Alphaproteobacteria;Rickettsiales	2	0.001

After bioinformatics analysis, all OTUs – except one attributed to the Rickettsiales order—had a 100% match with the database. When BLAST against it, the best alignment was with an uncultured bacterium clone (MH977830.1), with 97% query cover and 7 bp difference.

Regarding read abundance, *Lactobacillus kefiranofaciens* was the most present strain (21.96%), followed by *Lactobacillus kefiri* (0.2%) and *Acetobacter fabarum* (0.17%). Traces of *Lactococcus lactis* (0.004%) and Rickettsiales (0.001%) were also found.

To exclude the effects of microbiome interaction and analyze only the metabolite effects from kefir, a series of liquid–liquid partitioning was performed. From there, four organic fractions were obtained through liquid–liquid partitioning. Each fraction was named after its respective solvent. In polarity increasing order, we obtained: hexane (Hex), dichloromethane (DCM), ethyl acetate (EtOAc) and n-butanol (But-OH) fractions. Extraction yield increased with solvent polarity: n-butanol (ButOH) and ethyl acetate fraction (EtOAc) presented higher yields (8.71% and 3.11%) than dichloromethane (DCM) and hexane fractions (Hex) (1.37% and 0.50% respectively) (Table 2).

**Table 2:** Liquid–liquid partitioning yield from kefir methanolic extract, starting from 100 g of lyophilized kefir (fermented preparation).

Fraction	Solvent	Obtained weight (g)	Yield (%)
Hex	Hexane	0.1334	0.5053
DCM	Dichloromethane	0.3634	1.3765
EtOAc	Ethyl acetate	0.8226	3.1159
ButOH	N-butanol	2.3012	8.7136

Each fraction had its metabolites characterized through GC–MS analysis, in which 696 unique compounds were found (Supplementary Table 1). From those, 117 molecules were present in all four fractions, with 156 exclusive ones in Hexane, 66 in DCM, 47 in EtOAc, and 58 in ButOH. Principal Component Analysis (PCA) was able to discriminate all fractions, with Hexane having the most distinct metabolomic profile (Supplementary Figure S2).

### AD-like model validation

Before evaluating kefir’s effect in the amyloidogenic pathway, we validated our *D. melanogaster* AD-like model accessing neurodegeneration through amyloid quantification, flies’ climbing ability evaluation and histopathological analysis.

### Amyloid quantification

**Figure 1:**
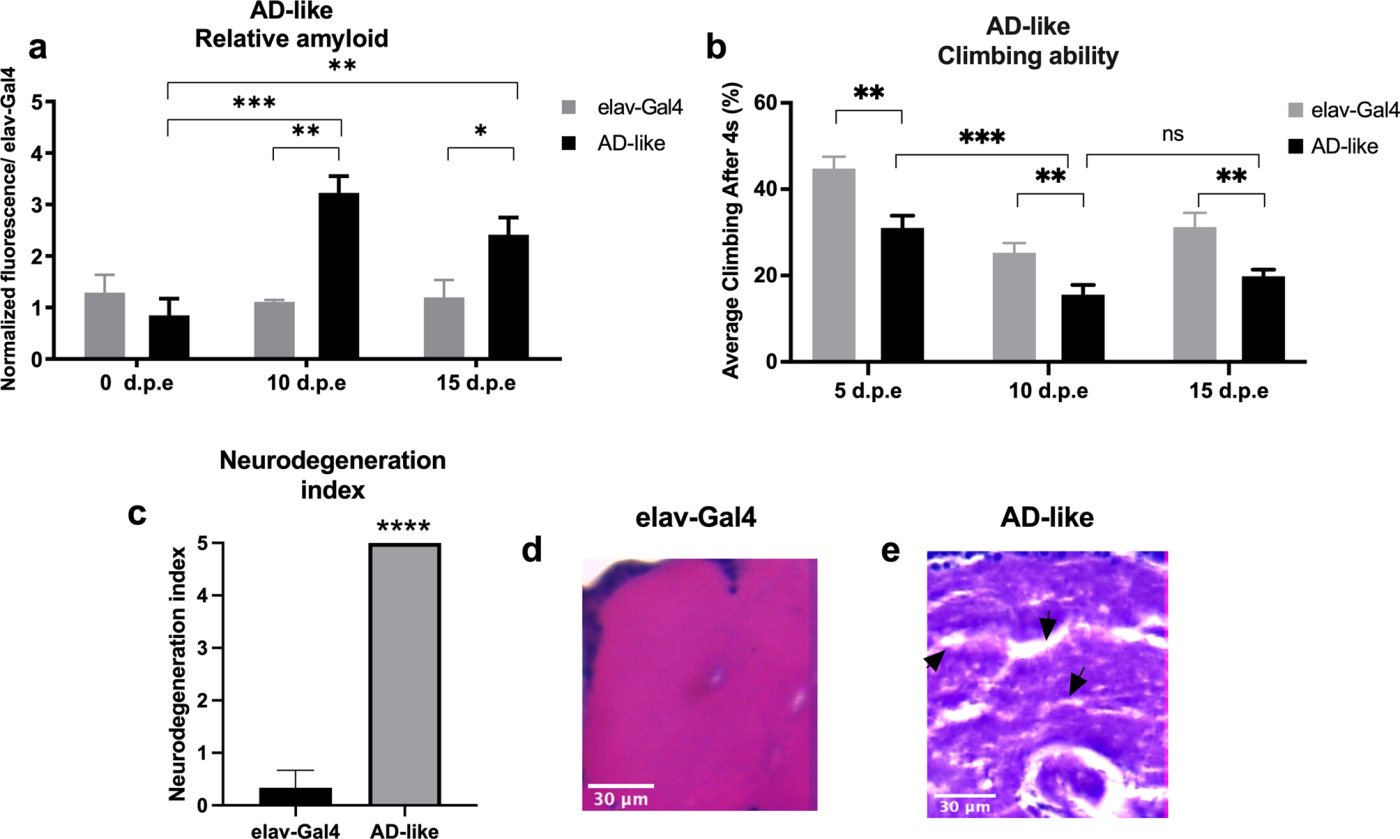
AD-like flies show a degenerative phenotype. (**a**) Amyloid quantification through Thioflavin aggregation assay. AD-like flies presented a higher amyloid content when compared to control flies (elav-Gal4) at 10- and 15-days post eclosion (d.p.e.) (n = 20 in each genotype). (**b**) Climbing ability. AD-like flies presented a lower climbing ability in the RING assay than the control genotype at 5–8, 10–13, and 15–18 days post eclosion. AD-like flies present a climbing ability decrease when comparing flies at 5–8 and 10–13 d.p.e. (n ≥ 180 in each genotype). (**c**) Neurodegeneration index of elav-Gal4 (control) and AD-like flies based on histopathology analysis, according to vacuolar lesions. 0 indicates no lesions, and 5 indicates neurodegenerative phenotype (n = 10, at 10 days after treatment). Data are shown as the mean ± S.E.M. The statistical significance is indicated as ** for P < 0.01 and *** for P < 0.001 (two-tailed ANOVA). Illustrative images of (**d**) elav-Gal4 and (**e**) AD-like flies’ histopathology. Arrows indicate vacuolar lesions.

Amyloid quantification is a direct method to verify functional expression of human BACE and APP. For that, a modified protocol from Westfall et al^28^ was implemented. We used Thioflavin T (ThT)—a benzothiazole dye that exhibits enhanced fluorescence upon binding to amyloid fibrils^29^—to quantify the amyloid content of AD-like flies (Fig. 1a).

AD-like flies had higher amyloid content than control flies both at 10 (P = 0,0011) and 15 days post eclosion (d.p.e.) (P = 0,0463), suggesting that at 0 days post eclosion there is still not a significant amyloid content being produced through the human BACE cleavage of human APP. There is an increase in amyloid fibrils when comparing flies at 0 to 10 days post eclosion (d.p.e.) (P = 0,0067), but it remains stable from 10 to 15 d.p.e.

### Rapid Iterative negative geotaxis (RING) assay

The Rapid Iterative Negative Geotaxis (RING) assay was used to measure motor reflex decline related to neurodegeneration, in here observed as the fly climbing ability. As expected, already at 5–8 d.p.e. AD-like flies presented a decline in climbing ability (Fig. 1b, P = 0,0024) in relation to control elav-Gal4 flies, which persisted at 10–13 (P = 0,0013) and 15–18 d.p.e (P = 0,0083). However, when looking at AD-like flies climbing ability though time, there is a significative decay in its climbing ability from 5–8 to 10–13 d.p.e. (P = 0.001), but not from 10–13 to 15–18 d.p.e. Therefore, AD-like flies will only be assayed on the first two trial ages.

### Histopathological analysis

To further verify the neurodegenerative phenotype of AD-like flies, histopathological analysis was performed at 10–13 d.p.e flies, with elav-Gal4 as a control. A neurodegeneration index from 0 to 5 was created according to vacuolar lesions in the central brain (Fig. 1c), being 0 no degeneration (as seen in elav-Gal4 flies, Fig. 1d) and 5, severe degeneration (as seen in AD-like flies, Fig. 1e). With these results, we confirm that the expression of human BACE and APP pan-neuronally induces a neurodegenerative phenotype at 10 d.p.e., and validate our model.

### Treatments

#### In natura kefir treatment

**Figure 2:**
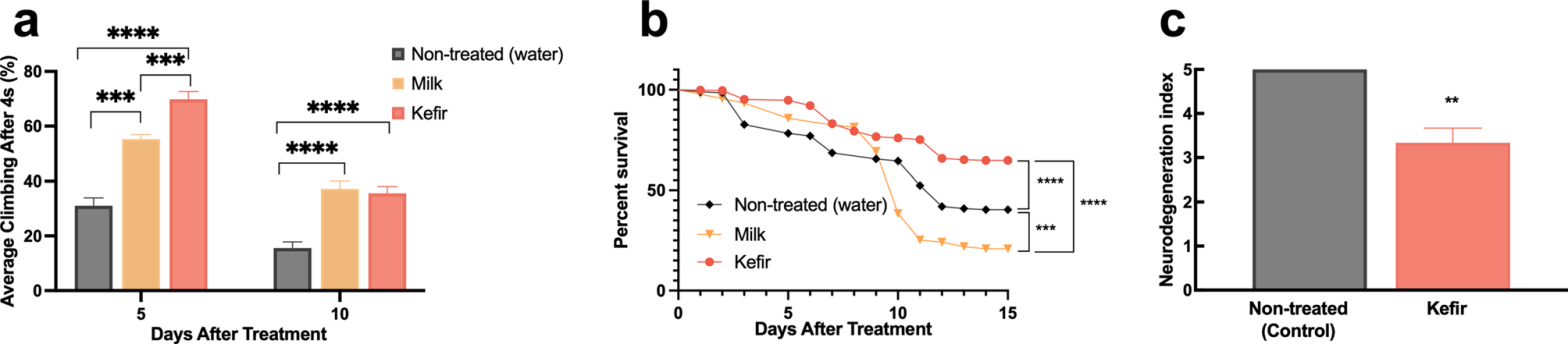
(**a**) Survival rate of non-treated, kefir, and milk-treated AD-like flies (n ≥ 90 in each group). The statistical significance is indicated as *** for P < 0.001 and **** for P < 0.0001 (log-rank, Mantel–Cox test). (**b**) AD-like flies climbing ability after kefir and milk treatment at 5 and 10 days after treatment (d.a.t.). Kefir-treated flies performed better than control at both tested ages, and better than milk-treated flies at 5 d.a.t.. At 10 d.a.t., flies treated with milk or kefir showed an equivalent performance. Data are shown as the mean ± S.E.M. n ≥ 90 in each group. (**c**) Neurodegenerative index of non-treated (control) and kefir treated AD-like flies (n = 10, 10 days after treatment). The statistical significance is indicated as ** for P < 0.01, *** for P < 0.001, **** for P < 0.0001 and (Unpaired two-tailed t-test).

Before investigating the effects of its metabolites, in natura kefir effects were accessed in AD-like flies. Control, fed with non-treated food, AD-like flies displayed a high mortality rate within the first two weeks of life, with only 40.4% of the flies alive (Fig. 2a). Kefir treatment improved the survival rate of these flies (P < 0.0001)—64.71% of kefir treated flies were still alive in the same period. This improvement was due to properties exclusive to kefir, as flies treated with non-fermented milk had a lower survival rate than control (P = 0.006), with only 20.8% of alive flies within the same period.

Through the RING assay, we verified that at 5 days after treatment (d.a.t.) AD-like flies fed with kefir displayed a higher climbing ability than both control (P < 0.0001) and flies treated with milk (P < 0.001) (Fig. 2b). At 10 d.a.t. this improvement in kefir treated flies is still present in relation to control flies (P < 0.0001), which is also shown in the histopathological analysis, where flies showed lower vacuolar lesions (P = 0,0037) (Fig. 2c, Supplementary Figure S3).

### Kefir fractions treatment

To verify if the improvements found in the neurodegenerative phenotype after kefir treatment was exclusive to microbiome interactions, or if it could also be achieved through isolated metabolites, we proceeded with testing cell and peptide free fractions. For each fraction, Tween80 0.01% was used as a vehicle, and three concentrations were tested: 0.1, 0.25 and 0.5 mg/mL.

**Figure 3:**
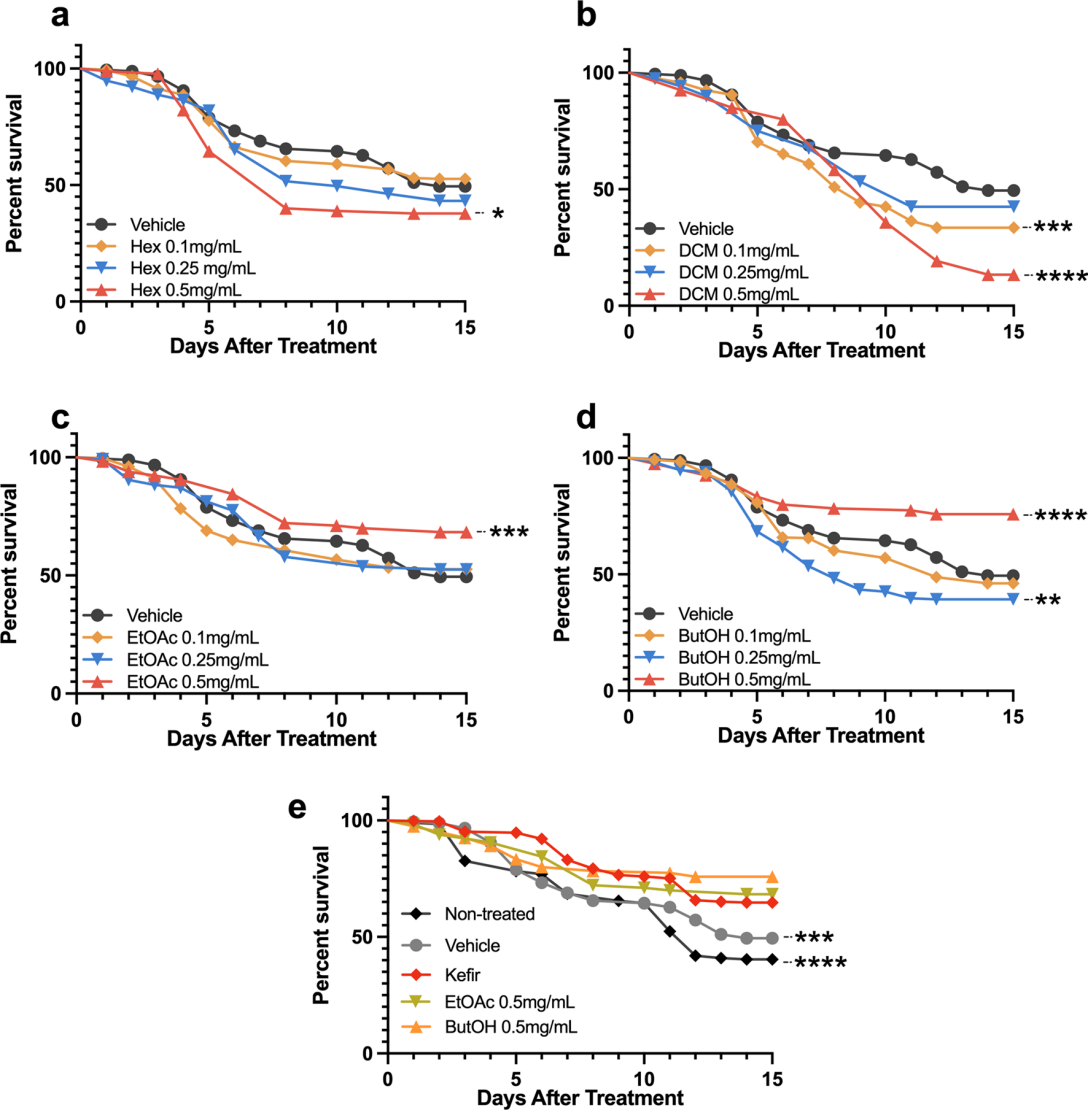
Survival rate of AD-like flies after treatment with (**a**) hexane, (**b**) dichloromethane, (**c**) ethyl acetate or (**d**) n-butanol fractions from kefir’s methanolic extract. (n ≥ 90 in each group). (**e**) Best performance fractions (ButOH and EtOAc 0.5 mg/mL) were compared to kefir-treated flies. The statistical significance is indicated as * for P < 0.05, ** for P < 0.01, *** for P < 0.001, and **** for P < 0.0001 (log-rank, Mantel–Cox test).

Vehicle and non-treated flies showed no difference in survival. On the other way, some fractions had a toxic effect on AD-like flies, with those displaying a lower survival rate in relation to vehicle-fed ones in the analyzed period. This happened for flies treated with Hex 0.5 mg/mL (P = 0,0290), DCM 0.1 and 0.5 mg/mL (P = 0,0006 and P < 0.0001, respectively), plus ButOH 0.25 mg/mL (P = 0,0084) (Fig. 3). Despite that, survival rate was improved on flies treated with EtOAc 0.5 mg/mL (P = 0.0005) and ButOH 0.5 mg/mL (P < 0.0001), which improved AD-like flies’ survival rate as much as the kefir treatment (Fig. 3e).

**Figure 4:**
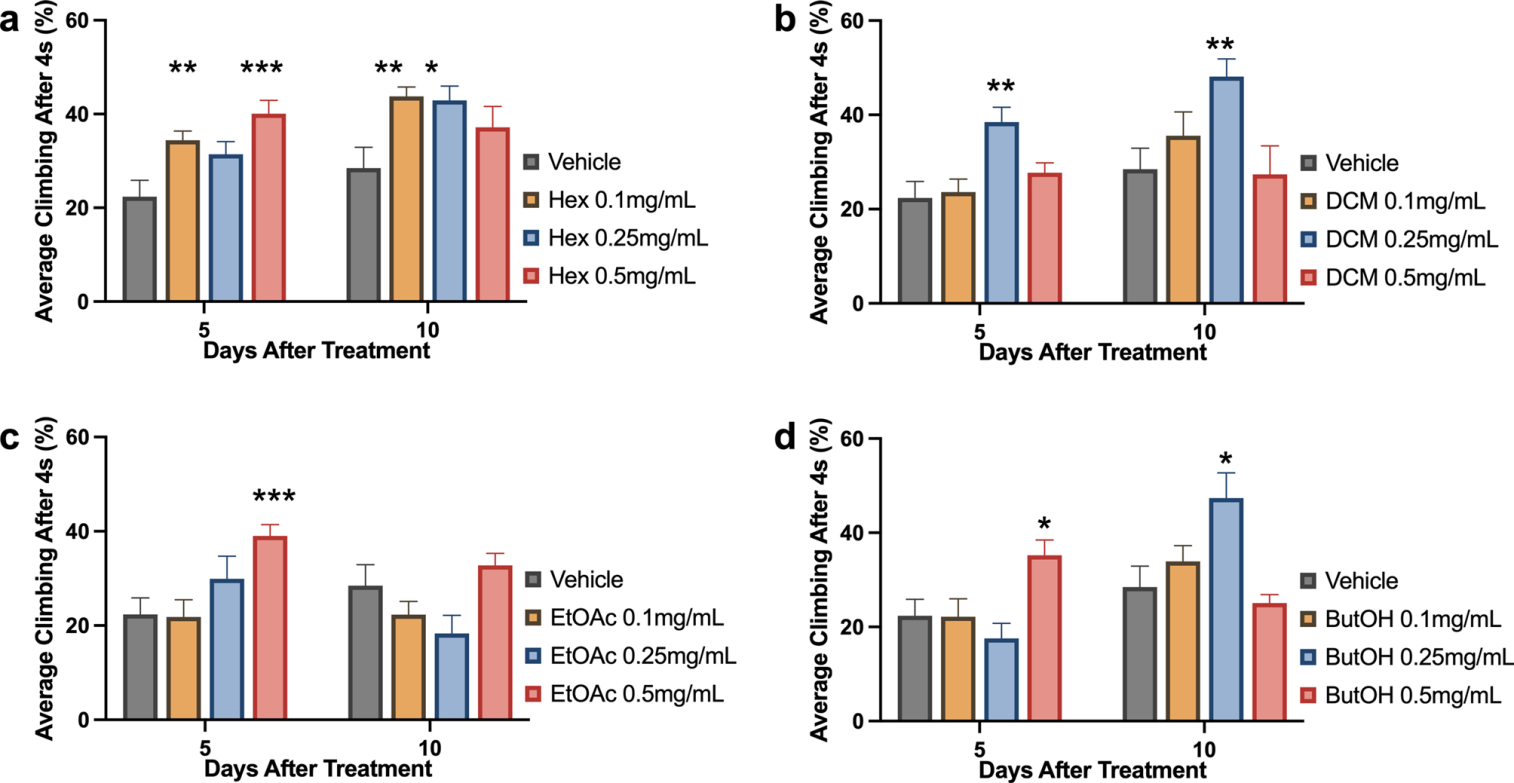
AD-like flies climbing ability after 5 and 10 days of treatment with Tween 0.01% (used as a vehicle) or either (**a**) hexane, (**b**) dichloromethane, (**c**) ethyl acetate or (**d**) n-butanol fractions at 0.1, 0.25 and 0.5 mg/mL. Data are shown as the mean ± S.E.M. (n ≥ 90 in each treatment). The statistical significance is indicated as * indicates P < 0.05, ** P < 0.01 and *** P < 0.001 in comparison to vehicle treated flies (Unpaired two-tailed t-test).

On the RING assay, vehicle treatment also did not change flies climbing ability in relation to control flies (fed with non-treatment food) both at 5 and 10 d.a.t. (data not shown). At 5 d.a.t., treatment with at least one concentration of each fraction improved AD-like flies climbing ability when compared with the ones treated only with vehicle (Fig. 4). Flies treated with DCM fraction performed the best on the RING assay when the 0.25 mg/mL concentration was used (P = 0,0051), while for both EtOAc and ButOH the best concentration was 0.5 mg/mL (P = 0,0006 and P = 0,0124, respectively). Two concentrations of Hex fraction increased AD-like flies climbing ability: 0.1 (P = 0,0096) and 0.5 mg/mL (P = 0.0009), being the last, the most efficient.

At 10 d.a.t., EtOAc-treated flies did not perform better than vehicle-fed ones. Flies treated with Hex 0.1 mg/mL and DCM 0.25 mg/mL maintained a better climbing ability performance than vehicle-treated flies (P = 0,0019 and P < 0,0001). Performance was improved (in relation to vehicle) for flies treated with ButOH and Hex at 0.25 mg/mL (P < 0.0001 and P = 0,0421)—rather than 0.5 mg/mL, which enabled the best performance at 5 d.a.t. for both fractions in this assay.

**Figure 5:**
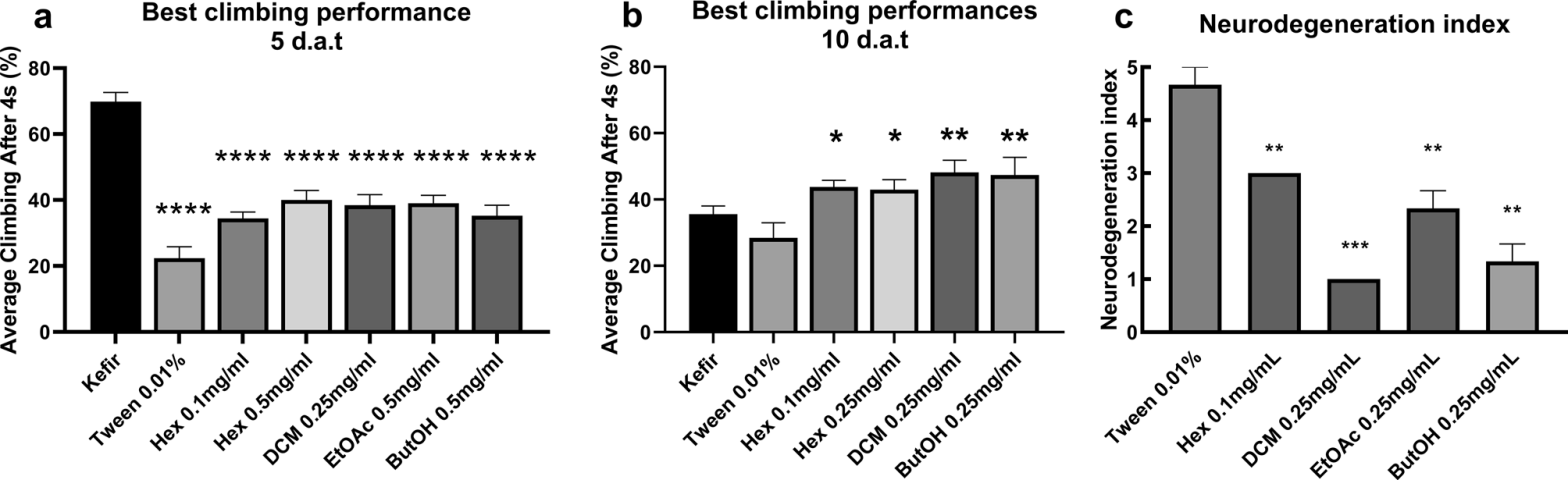
Average climbing ability of flies treated with best performance fractions and kefir, as well as control and vehicle after (**a**) 5 or (**b**) 10 days after treatment. Data are shown as the mean ± S.E.M. n ≥ 90 in each treatment. The statistical significance is indicated in relation to kefir group as * indicates P < 0.05, ** P < 0.01 and *** P < 0.001 (Unpaired two-tailed t-test). (**c**) Neurodegenerative index of flies treated with all four fractions 10 days after treatment, at its best performance concentration (n = 10). The statistical significance is indicated in relation to Tween 0.01% group as ** indicates P < 0.01 and *** P < 0.001 (Unpaired two-tailed t-test).

Comparing the best performance fractions with kefir treatment, at 5 d.a.t., kefir-treated flies had a higher climbing ability than the ones treated with fractions (P < 0.0001) (Fig. 5a). At 10 d.a.t., all treatments that improved AD-like flies climbing ability performed better than kefir-treated flies: Hex 0.1 (P = 0,0145) and 0.25 mg/mL (P = 0,1253); DMC 0.25 mg/mL (P = 0,0064) and ButOH 0.25 mg/mL (P = 0,0298) (Fig. 5b). Beyond, on histopathological analysis, all fractions attenuated AD-like vacuolar lesions when compared to vehicle treated flies (Fig. 5c, Supplementary Figure S3). Flies treated with DCM 0.25 mg/mL showed the lowest neurodegenerative index amongst all tested fractions (P = 0,0004). ButOH treatment at 0.25 mg/mL also brought neurodegeneration to low (P = 0,0021), followed by moderate neurodegeneration after EtOAc 0.25 mg/mL (P = 0,0039) and Hex 0.1 mg/mL (P = 0,0037) treatment.

**Figure 6:**
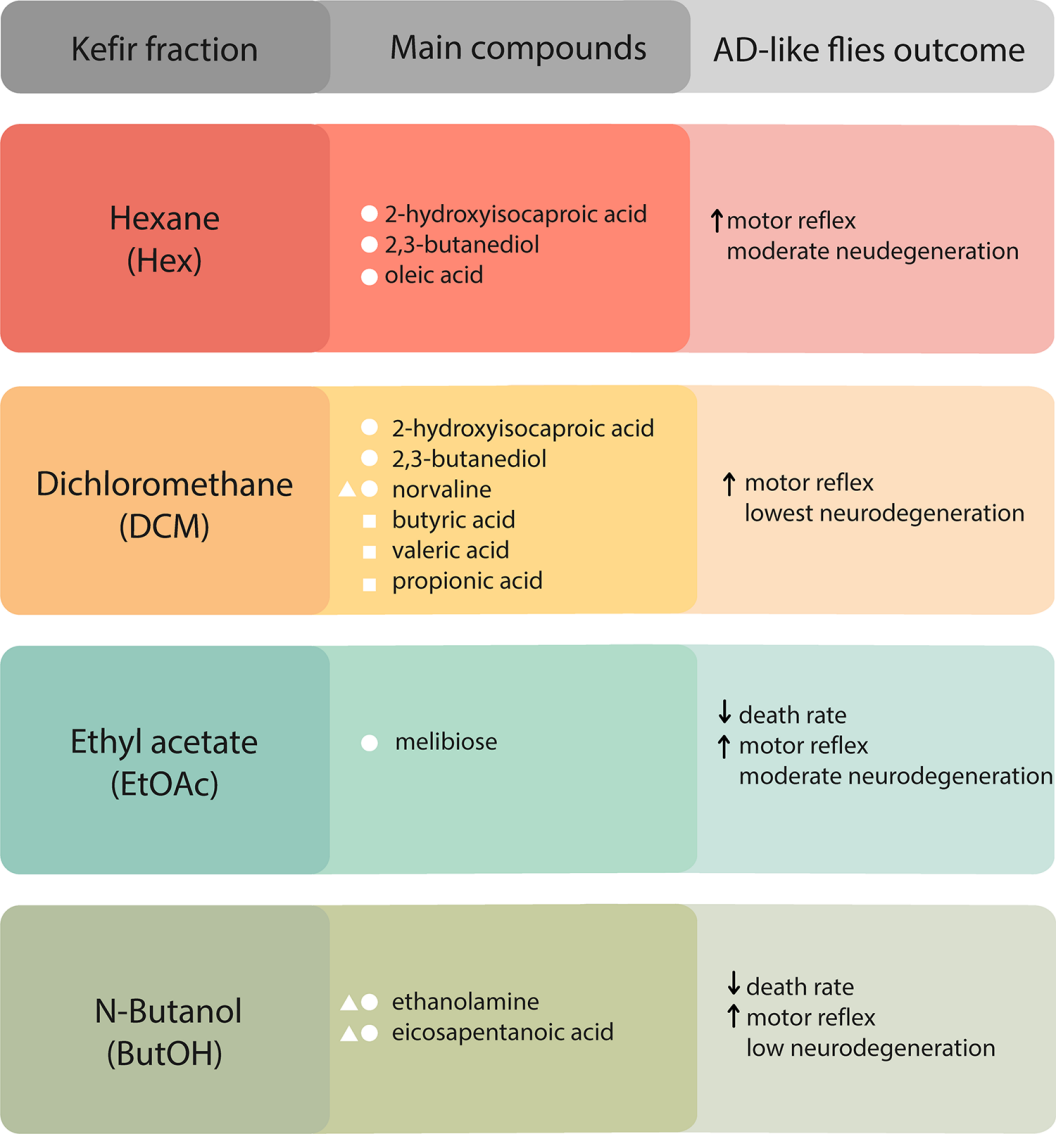
Brief summary of the main compound of each kefir fraction and of the outcome of treated AD-like flies. The compounds were identified by CG-MS, the motor reflex was evaluated by climbing assay, and the neurodegeneration index was evaluated by histopathological analysis. Upwards arrows indicate improvement in the evaluated outcome and downwards arrows indicate its decrease. White symbols indicate the activity of compounds exclusive to each. Squares indicate SCAFS, balls indicate compounds with anti-inflammatory activity, and triangles indicate anti-oxidants.

The main findings of this work have been summarized in Fig. 6.

## Discussion

Kefir microbiota can vary depending on the grains’ geographical origin, storage, and fermentation parameters (used milk type, grain-milk ratio, and temperature)^30–32^. Therefore, characterizing our sample’s bacterial microbiota—on composition and abundance—was needed to provide its fingerprint. *Lactobacillus kefiranofaciens* and *Lactobacillus kefiri* were the most present in kefir, followed by *Lactococcus lactis* and *Acetobacter fabarum*. A similar profile has also been shown in kefir samples from France, Ireland and the United Kingdom^33^, Belgium^34^, Malaysia^35^, Italy^36^, and Brazil^37^.

Kefir’s health improving effects have been demonstrated when used in natura^19,38–41^, as cell-free fraction^42–45^ and as purified peptides^46–50^. However, no previous studies have characterized kefir metabolic composition, or investigated the biological effects of its peptide and cell-free fraction. This made us interested in evaluating kefir metabolic fraction as a treatment to improve AD-like flies’ degenerative phenotype.

For that, fermented kefir underwent methanol extraction, which precipitates microorganisms and peptides^52^. Due to kefir’s microbiological diversity, we hypothesized it would reflect on a vast quantity of secondary metabolites. This way, we decided to partition this extract with four organic solvents with increasing polarity, to obtain a broader distribution of molecules and test their effect in the amyloidogenic pathway in *D. melanogaster*.

Our first step was to validate AD-like flies, which express APP and BACE by using a pan neuron driver (elav-Gal4). AD-like flies showed a higher amyloid content than control flies at 10- and 15-days post eclosion, which correlates with a decrease in climbing ability at the same age. Plus, within this interval, AD-like flies have a higher mortality rate than control flies and presence of vacuolar lesions at 10 d.p.e.. These phenotypical changes have been previously reported in AD-like flies, indicating the suitability of our model^53,54^.

After kefir treatment, we assayed flies’ survival and locomotor activity, as well as its neurodegeneration index through histology analysis. Treatment with kefir *in natura* improved AD-like flies’ survival rate, climbing ability and attenuated vacuolar lesions from severe to moderate-low, which correlates with previous studies, in which kefir has shown to improve learning and memory^21^, and reduce oxidative stress and inflammation^51^ in AD patients.

Regarding the effects of kefir fractions, non-polar fractions (hexane and dichloromethane) improved fly climbing in both tested ages—generating a better outcome than kefir at 10 d.a.t.. Instead, more polar fractions—ethyl acetate and n-butanol—had a higher impact in improving AD-like flies’ survival.

In the histopathological analysis, the most efficient fraction to attenuate vacuolar lesions was dichloromethane, followed by n-butanol (both from severe to low). However, Hexane and ethyl acetate treated flies had a reduction of vacuolar lesions from severe to moderate.

When investigating each fraction’s metabolome, we found 117 compounds shared by all fractions. Among those shared compounds, we found the short-chain fatty acids (SCFAs): levulinic acid and linoleic acid. SCFAs are markedly downregulated in AD models in *D. melanogaster*^64^ and in mice^65^, and treatment with these fatty acids has been previously shown to inhibit Aβ aggregation in vitro^66^. Linoleic acid, specifically, has been shown to inhibit BACE activity in silico^67^ and to reduce Aβ oligomerization related neuronal death^68^. Beyond, we highlight compounds with antioxidant (3-pyridinol^55^, methyl linoleate^56^, oxalic acid^57^, d-allose^58^, 1-dodecanol^59^) and anti-inflammatory activity (ethyl linoleate, methyl linoleanate^60^, lauric acid^61^) or even both (1-octadecanol^62^, heptocosane^63^). Finally, stearic acid and heneicosanoic acid have been described to inhibit β-amyloid aggregation and acetylcholinesterase *in silico*^69^.

The presence of these compounds with antioxidant, anti-inflammatory, and anti-BACE activity in all fractions would explain the improvements seen in the evaluated AD-like characteristics. More specifically, we also investigated relevant compounds that were exclusive to each fraction. Within hexane fraction, we highlight oleic acid, which has been reported to inhibit BACE in silico^67^ and to ameliorate amyloidosis in vitro and in mice models of Alzheimer’s disease^70^. Within dichloromethane, norvaline was present, which has previously shown to act both on anti-inflammatory and antioxidative processes in a mouse model of Alzheimer’s Disease^71^. Beyond, more SCFAs were detected, as butyric acid, valeric acid and propionic acids, which have been reported to inhibit Aβ aggregation in vitro^66^. Within the ethyl acetate fraction, melibiose was present, which has been listed as a promise for polyQ-mediated neurodegenerative disease treatment^73^ as AD for its anti-inflammatory mechanisms, which have improved Parkinson-related motor behavior in mice^72^. Last, within the n-butanol fraction, both ethanolamine and eicosapentaenoic acid were present. This combination has been described to attenuate oxidative stress, neuroinflammation, apoptosis, and Aβ-induced neurotoxicity in AD-model mice^74^.

In conclusion, this present work investigated the effect of kefir *in natura* and its metabolic fractions in the AD’s amyloidogenic pathway. Kefir microbiota composition was determined through 16S sequencing, finding *Lactobacillus kefiranofaciens* as its most abundant species and detecting one yet unknown bacterial species. To our knowledge, this is the first report comparing the effect of a probiotic *in natura* and its metabolic fractions, as well as its metabolome description. In an overview, n-butanol fraction performed the best: it improved AD-like flies’ climbing ability, and attenuated vacuolar lesions, without compromising its survival—which happened with flies treated with dichloromethane. The metabolomic identification of these molecules—and correlation to their described beneficial effects within AD-related mechanisms—helps us understand more about kefir effects in the amyloidogenic pathway.

## Methods

### Kefir characterization

Kefir grains were received as a donation in Uberlândia, Brazil. The fermented product—kefir—was obtained by inoculating kefir grains (4% m/v) in pasteurized whole cow milk. The fermentation process went for 24 h at room temperature in a glass container covered with cloth to avoid contamination. Then, kefir grains were filtered, and the fermentation product was used for fly treatments. Exceeding grains were inoculated in milk (20% glycerol) and were kept at -20ºC for further experiments.

### Next-generation sequencing

To investigate its bacterial composition and generate an identity of our product, kefir grains together with its fermented product were sequenced. The genomic DNA was purified according to the BGI Americas in-house protocol, and its integrity was tested using 1% agarose gel electrophoresis. Sample concentration was tested using Qubit Fluorometer (Invitrogen).

For library construction, 30 ng of DNA and fusion primers were used to configure PCR for 16S-v4 regions (BGI Americas in-house protocol). After PCR, DNA was purified using Agencourt AMPure XP beads (DNA/bead ratio of 1). The library was qualified with the Agilent 2100 bioanalyzer (Agilent Technologies), and paired-end sequencing was done on Hiseq 2500 (Ilumina), using the MiSeq-PE250 sequencing strategy (MiSeq Reagent Kit).

For obtaining more accurate and reliable results, raw data was pre-processed by removing: reads with a lower average quality of 20 over 25 bp—based on the phred algorithm^75^; trimmed reads with less than 75% of their original length; reads contaminated by adapters (with 15 bp overlapped); and reads with low complexity (with 10 consecutive same base). Plus, if the two paired-end reads overlapped (minimum of 15 bp overlap), the consensus sequence was generated by FLASH (Fast Length Adjustment of Short reads, v1.2.11)^76^.

### 16S data analysis

To analyze community patterns, all clean the tags were clustered to OTUs (Operational Taxonomic Units) using USEARCH (v7.0.1090)^77^. The tags were clustered into OTUs with a 97% threshold by using UPARSE^78^, and unique, representative OTU sequences were obtained. Chimeras were then filtered out by using UCHIME(v4.2.40)^79^. Finally, chimeras were screened by mapping to Gold Database (v20110519)^80^ and to UNITE (v20140703)^81^.

All OUT tags were mapped to representative sequences using USEARCH GLOBAL, and were taxonomically classified using Ribosomal Database Project (RDP) Classifier v.2.2^82^ (cutoff = 0.6). Bacterial 16S rDNA were annotated using Greengene database (v201305)^83^ and BLAST searched against the National Center for Biotechnology Information (NCBI) nucleotide collection. Species were qualified when query cover was 100%.

### Kefir fractions

To obtain a cell and peptides free metabolite fraction, the fermented product was frozen at − 20 °C overnight and then lyophilized (L101, Liobras, SP, Brazil) for three days. Lyophilized material (100 g) was solubilized on 150 mL of methanol 80% for 15 min. The liquid phase was obtained through filter paper separation, following to liquid–liquid partitioning. In increasing polarity, hexane, dichloromethane, ethyl acetate, and n-butanol solvents were used. For each solvent, 200 mL were used and the process was repeated four times. The solvents were removed using a rotary evaporator (Buchi Rotavapor R-210, Flawil, Switzerland) and the fractions were left to evaporate in a chemical hood for a week. The resulting fractions were frozen overnight and lyophilized to remove the remaining water.

When evaluating kefir’s metabolite, the usage of a vehicle was needed to improve fractions solubility. For that, Tween 80 successfully solubilized for all four fractions with the same concentration. Plus, it had the advantage of not influencing AD-like phenotype. Tween treatment displayed no effect on flies survival, climbing ability, or histopathological analysis. This is important since other vehicles, as DMSO and PEG, have shown neuroprotective effects or even CSN modulation^84–88^.

### Metabolomics

To characterize its composition via GC–MS, an adapted protocol from Fiehn^89^ was used. Each fraction (sample) was solubilized in its respective solvent. Samples were split in 5 aliquots and 5uL of internal standard D27 Myristic acid (3 mg/mL) was added to each aliquot. Samples were dried in SpeedVac Vacuum (Thermo) for 18 h.

For derivatization, dried metabolites were solubilized in 20μL of methoxylamine (40 mg/mL in pyridine), spiked with 3μL of FAME (Fatty acid methyl ester–Sigma-Aldrich) and incubated at 25 °C during 16 h at 650 rpm agitation. Then, 90μL of MSTFA with 1%TMCS was added. Samples were incubated for 1 h at 25 °C, followed by centrifugation at 15800 g for 10 min at room temperature. After derivatization, 1μL of each sample was injected randomly into an Agilent 7890B GC system operated in splitless mode, in triplicate. A DB5-MS + 10 m Duraguard capillary column (Agilent #122-5532G) within which helium carrier gas flowed at a rate of 0.82 mL min^−1^, was applied for metabolite separation. The injector temperature was set at 250 °C. The column temperature was held at 60 °C for 1 min, and then increased to 310 °C at a rate of 10 °C/min during 37 min. The column effluent was introduced into the ion source of an Agilent 5977A mass selective detector. The detector operated in the electron impact ionization mode (70 eV) and mass spectra were recorded after a solvent delay of 6.5 min with 2.9 scans per second, starting at mass 50 and ending at mass 550, with step size of 0.1 m/z. The MS quadrupole temperature was set at 180 °C and the ion source temperature was set at 280 °C.

Data were deconvoluted with RT size window of 75 and 100, SNR threshold of 1, extraction window m/z delta of 0.3 AMU on left and 0.7 on right. Identification of compounds was made comparing the mass spectra and retention time (RT) of all detected compounds with the Agilent Fiehn GC/MS Metabolomics RTL Library (version A.02.02). The metabolites that were not identified in the Agilent Fiehn GC/MS Metabolomics RTL Library were searched in the National Institute of Standards and Technology (NIST) library 11 (2014) using Unknowns—Agilent MassHunter Workstation Quantitative Analysis (version B.06.00).

Data consistency was checked based on the identification of Internal Standard (Myristic Acid D27) among samples, considering a coefficient variation of metabolites intensity lower than 30% among the 3 technical replicates. Only metabolites present in at least 3 of 5 samples from each group were submitted to statistical analysis. Metabolites were analyzed using the MetaboAnalyst online platform. Missing values were substituted by 1/5 of lower value in the table and data was normalized using auto-scaling method and log transformation.

### *D. melanogaster* stocks

Flies were reared with standard cornmeal medium (soy powder 0,01%, glucose 7,2%, agar 0,6%, cornmeal 0,073%, yeast 0,018%, nipagin 0,06% and acid solution 0,05% m/v) and kept in a 12–12 h light/dark cycle incubator, at 25ºC.

To generate flies expressing human BACE and APP pan-neuronally, individuals from elav-Gal4 (Bloomington Stock Center #458) and UAS-BACE,UAS-APP (#33,797) strains were anesthetized and sorted according to sex. Elav-Gal4 female virgins—with a visible meconium—and UAS-BACE,UAS-APP males and were crossed. The resulting F1 was sorted through while in pupae stage: the ones exhibiting tubby phenotype were discarded. These steps ensured the resulting individuals would be elav-Gal4; ;UAS-BACE,UAS-APP, hereafter addressed as AD-like flies.

### Total amyloid quantification

To verify the amyloidogenic pathway from AD-like, total amyloid content was assessed using the Thioflavin T (ThT), a benzothiazole dye that exhibits enhanced fluorescence upon binding to amyloid fibrils^28,29^. Fly heads of elav-Gal4 (control) and AD-like flies (pool of 20 heads each, in triplicate) were collected, homogenized in Tris–EDTA-Triton buffer with protease inhibitor (cOmplete Lysis-M (Sigma)) and centrifuged. The supernatants were collected and used for amyloid quantification and Bradford protein dosage in technical triplicate. In a protocol modified from Westfall et al.^28^, supernatants were incubated with ThT working solution (20 µM) for 20 min under agitation. Fluorescence was measured at 450 nm excitation / 482 nm emission and normalized to ThT only samples. Total fluorescence was corrected to each sample’s total protein content and to elav-Gal4 (control flies) fluorescence levels. This additional step was needed since in vivo samples could present autofluorescence.

### Treatments

For all assays, AD-like flies were treated at 0–3 days after eclosion. Treatment food was prepared by adding 2 mL of freshly-prepared kefir or its fractions (hexane, dichloromethane, ethyl acetate or n-butanol) to 1 g of enriched mashed potato medium (75% instant mashed potato, 15% yeast extract, 9,3% glucose, and 0,07% nipagin). Food was changed every 2 days to ensure fresh treatment exposure. Beyond, water (control) and Tween80 0.01% (Sigma) (vehicle) were tested.

### Survival assay

In order to evaluate kefir (*in natura* and its fractions) toxicity, AD-flies survival rate was accessed. Male and female flies were treated as previously described and dead flies were counted every two days for 15 days. A total of 90 flies from each genotype and treatment were assayed. The mean lifespan was calculated through the Kaplan–Meier test on GraphPad Prism 8.0.2 software.

### Rapid iterative negative geotaxis (RING) assay

Since fly motor reflex is related to neurodegeneration in *D.* melanogaster, we accessed its climbing ability after treatments. For this, groups of 30 male control (elav-Gal4) and AD-like flies of each treatment (tested in triplicate) were transferred to clean vials and put in a custom 12-vials holder. Flies had their behavior accessed 5 and 10 days after treatment. Before testing, flies were exposed to light and kept in a silent environment for 20 min, in order to acclimate. The holder was then hit three times on the bench, and the flies were given 4 s to climb 5 cm. This was repeated five times. The procedure was recorded and the video analyzed using QuickTime Player 7.7.9 software. The average climbing percentage was calculated as the percentage of flies of each group that reached the 5 cm mark after 120 frames that the holder touched the bench.

### Histopathological analysis

Ten flies of each treatment group, at the best performed concentration in previous experiments were used. All flies were analyzed at 10 d.p.e. and elav-Gal4 was used as a control. The flies had its head transferred to 4% formaldehyde in sodium phosphate buffer 0.1 M pH 7.2 for 16 h at 4 °C. The samples were then dehydrated in a graded ethanol series (70, 80, 90, and 95%) and transferred to methanol for 16 h at 4 °C. After, they were embedded in HistoResin (Leica) and 2 µm thick slices were stained with hematoxylin and eosin, analyzed and photographed with a light photomicroscope. Neuropile images were used to calculate the neurodegenerative index—as normal to low, moderate, or severe—according to vacuolar lesions^90^.

### Statistical analysis

Obtained data distribution for each analyzed group within each experiment was evaluated as either parametric or non-parametric through the D’Agostino&Pearson test. Groups were compared through a t test with a stablished significance level of P < 0.05. Analysis was performed using the software GraphPad Prism 8.

## Supplementary Information

Supplementary Information 1.

Supplementary Information 2.

## Data Availability

The raw data generated from 16S-based microbiome NGS profiling have been deposited in the BioProject database (NCBI), under the Submission ID: SUB9542632 and the BioProject ID: PRJNA725245. The other datasets generated during and/or analysed during the current study are available from the corresponding author on request.

## References

[1] Livingston G (2020). Dementia prevention, intervention, and care: 2020 report of the Lancet Commission. Lancet.

[2] Lane CA, Hardy J, Schott JM (2018). Alzheimer’s disease. Eur. J. Neurol..

[3] Hardy J, Selokoe D (2002). The amyloid hypothesis of Alzheimer ’s disease. Amyloid Int. J. Exp. Clin. Investig..

[4] Bhattacharjee S, Lukiw W (2013). Alzheimer’s disease and the microbiome. Front. Cell. Neurosci..

[5] Bonfili L (2017). Microbiota modulation counteracts Alzheimer’s disease progression influencing neuronal proteolysis and gut hormones plasma levels. Sci. Rep..

[6] Sun M (2020). A Review of the Brain-Gut-Microbiome Axis and the Potential Role of Microbiota in Alzheimer’s Disease. J. Alzheimers. Dis..

[7] Morales R (2010). Molecular cross talk between misfolded proteins in animal models of Alzheimer’s and prion diseases. J. Neurosci..

[8] Gao Q (2019). Decreased levels of circulating trimethylamine N-oxide alleviate cognitive and pathological deterioration in transgenic mice: A potential therapeutic approach for Alzheimer’s disease. Aging.

[9] Erickson MA (2012). Lipopolysaccharide impairs amyloid beta efflux from brain: Altered vascular sequestration, cerebrospinal fluid reabsorption, peripheral clearance and transporter function at the blood–brain barrier. J. Neuroinflammation.

[10] Westfall S (2017). Microbiome, probiotics and neurodegenerative diseases: Deciphering the gut brain axis. Cell. Mol. Life Sci..

[11] Plessas Stravos (2016). Microbiological exploration of different types of kefir grains. Fermentation.

[12] Dong J, Liu B, Jiang T, Liu Y, Chen L (2018). The biofilm hypothesis: The formation mechanism of Tibetan kefir grains. Int. J. Dairy Technol..

[13] Amorim FG (2019). Identification of new bioactive peptides from Kefir milk through proteopeptidomics: Bioprospection of antihypertensive molecules. Food Chem..

[14] Kim DH, Jeong D, Kim H, Seo KH (2019). Modern perspectives on the health benefits of kefir in next generation sequencing era: Improvement of the host gut microbiota. Crit. Rev. Food Sci. Nutr..

[15] Cenesiz S, Devrim AK, Kamber U, Sozmen M (2008). The effect of kefir on glutathione (GSH), malondialdehyde (MDA) and nitric oxide (NO) levels in mice with colonic abnormal crypt formation (ACF) induced by azoxymethane (AOM). Dtsch. Tierarztl. Wochenschr..

[16] Chen Z (2015). Chemical and physical characteristics and antioxidant activities of the exopolysaccharide produced by Tibetan ke fi r grains during milk fermentation. Int. Dairy J..

[17] Rodrigues KL, Gaudino Caputo LR, Tavares Carvalho JC, Evangelista J, Schneedorf JM (2005). Antimicrobial and healing activity of kefir and kefiran extract. Int. J. Antimicrob. Agents.

[18] Hsieh H-H, Wang S-Y, Chen T-L, Huang Y-L, Chen M-J (2012). Effects of cow’s and goat’s milk as fermentation media on the microbial ecology of sugary kefir grains. Int. J. Food Microbiol..

[19] Anwar, M. M., Ali, O. S. M., Laila Ahmed, R., Badawi, A. M. & Eltablawy, N. A. The effect of using kefir grains and mesenchymal stem cells in LPS-induced Alzheimer’s disease neuroinflammatory model. *Neurobiol. Rev. electrónica* (2019).

[20] Anwar, M. M., Ali, O. S. M., Laila Ahmed, R., Badawi, A. M. & Eltablawy, N. A. Regulation of miRNA-124, nuclear factor-Kappa B and β-Catenin expression in response to novel therapeutic protocol in LPS induced Alzheimer’s disease in rats. *Bone* **1**, 17–19 (2018).

[21] Ton, A. M. M. *et al.* Oxidative Stress and Dementia in Alzheimer’s Patients: Effects of Synbiotic Supplementation. *Oxid. Med. Cell. Longev.* **2020**, (2020). 10.1155/2020/2638703 PMC7201593 32411323

[22] Tue NT, Dat TQ, Ly LL, Anh VD, Yoshida H (2020). Insights from Drosophila melanogaster model of Alzheimer’s disease. Front. Biosci. - Landmark.

[23] Jeon, Y., Lee, J. H., Choi, B., Won, S. Y. & Cho, K. S. Genetic dissection of Alzheimer’s disease using Drosophila models. *Int. J. Mol. Sci.* **21**, (2020). 10.3390/ijms21030884 PMC7037931 32019113

[24] McGurk L, Berson A, Bonini NM (2015). Drosophila as an in vivo model for human neurodegenerative disease. Genetics.

[25] Lenz S, Karsten P, Schulz JB, Voigt A (2013). Drosophila as a screening tool to study human neurodegenerative diseases. J. Neurochem..

[26] Westfall Susan (2018). A novel polyphenolic prebiotic and probiotic formulation have synergistic effects on the gut microbiota influencing Drosophila melanogaster physiology. Artificial Cells, Nanomedicine, and Biotechnology.

[27] Tan FHP (2020). Lactobacillus probiotics improved the gut microbiota profile of a Drosophila melanogaster Alzheimer’s disease model and alleviated neurodegeneration in the eye. Benef. Microbes.

[28] Westfall S, Lomis N, Prakash S (2019). A novel synbiotic delays Alzheimer’s disease onset via combinatorial gut-brain-axis signaling in Drosophila melanogaster. PLoS ONE.

[29] Khurana R (2005). Mechanism of thioflavin T binding to amyloid fibrils. J. Struct. Biol..

[30] Londero A, Hamet MF, De Antoni GL, Garrote GL, Abraham AG (2012). Kefir grains as a starter for whey fermentation at different temperatures: Chemical and microbiological characterisation. J. Dairy Res..

[31] Rosa DD (2017). Milk kefir: Nutritional, microbiological and health benefits. Nutr. Res. Rev..

[32] Nielsen B, Gürakan GC, Unlü G (2014). Kefir: A multifaceted fermented dairy product. Probiotics Antimicrob. Proteins.

[33] Walsh Aaron M. (2016). Microbial Succession and Flavor Production in the Fermented Dairy Beverage Kefir. Applied and Environmental Science.

[34] Korsak N (2015). Evaluation of the microbiota of kefir samples using metagenetic analysis targeting the 16S and 26S ribosomal DNA fragments. J. Dairy Sci..

[35] Zamberi NR (2016). 16S Metagenomic Microbial Composition Analysis of Kefir Grain using MEGAN and BaseSpace. Food Biotechnol..

[36] Garofalo C (2015). Bacteria and yeast microbiota in milk kefir grains from different Italian regions. Food Microbiol..

[37] Leite AMO (2012). Assessment of the microbial diversity of Brazilian kefir grains by PCR-DGGE and pyrosequencing analysis. Food Microbiol..

[38] Sharifi M (2017). Kefir: A powerful probiotics with anticancer properties. Med. Oncol..

[39] Maciel FR (2016). Immunomodulation and nitric oxide restoration by a probiotic and its activity in gut and peritoneal macrophages in diabetic rats. Clin. Nutr..

[40] El Golli-Bennour E, Timoumi R, Koroit M, Bacha H, Abid-Essefi S (2019). Protective effects of kefir against zearalenone toxicity mediated by oxidative stress in cultured HCT-116 cells. Toxicon.

[41] Vasquez EC, Aires R, Ton AMM, Amorim FG (2020). New insights on the beneficial effects of the probiotic kefir on vascular dysfunction in cardiovascular and neurodegenerative diseases. Curr. Pharm. Des..

[42] Taheur F. Ben (2020). Inhibitory effect of kefir on Aspergillus growth and mycotoxin production. Euro-Mediterranean J. Environ.

[43] Rajoka MSR (2019). Characterization and anti-tumor activity of exopolysaccharide produced by Lactobacillus kefiri isolated from Chinese kefir grains. J. Funct. Foods.

[44] Jalali F, Sharifi M, Salehi R (2016). Kefir induces apoptosis and inhibits cell proliferation in human acute erythroleukemia. Med. Oncol..

[45] Kim DH (2016). Antimicrobial activity of kefir against various food pathogens and spoilage bacteria. Korean J. Food Sci. Anim. Resour..

[46] Şanli T, Akal HC, Yetişemiyen A, Hayaloglu AA (2018). Influence of adjunct cultures on angiotensin-converting enzyme (ACE)-inhibitory activity, organic acid content and peptide profile of kefir. Int. J. Dairy Technol..

[47] Miao J (2016). Inhibitory effects of a novel antimicrobial peptide from kefir against Escherichia coli. Food Control.

[48] Quirós A, Hernández-Ledesma B, Ramos M, Amigo L, Recio I (2005). Angiotensin-converting enzyme inhibitory activity of peptides derived from caprine kefir. J. Dairy Sci..

[49] Kaur H (2020). Effects of Probiotic Supplementation on Short Chain Fatty Acids in the App NL-G-F Mouse Model of Alzheimer’s Disease. J. Alzheimer’s Dis..

[50] Tung YT (2018). Kefir Peptides Prevent Hyperlipidemia and Obesity in High-Fat-Diet-Induced Obese Rats via Lipid Metabolism Modulation. Mol. Nutr. Food Res..

[51] Akbari, E. *et al.* Effect of probiotic supplementation on cognitive function and metabolic status in Alzheimer’s disease: A randomized, double-blind and controlled trial. *Front. Aging Neurosci.* **8**, 2 (2016). 10.3389/fnagi.2016.00256 PMC5105117 27891089

[52] Gowda GAN, Raftery D (2014). Quantitating Metabolites in Protein Precipitated Serum Using NMR Spectroscopy..

[53] Wang X (2014). Effects of curcuminoids identified in rhizomes of Curcuma longa on BACE-1 inhibitory and behavioral activity and lifespan of Alzheimer’s disease Drosophila models. BMC Complement. Altern. Med..

[54] Chiu WYV, Koon AC, Ngo JCK, Chan HYE, Lau K-F (2017). GULP1/CED-6 ameliorates amyloid-β toxicity in a Drosophila model of Alzheimer’s disease. Oncotarget.

[55] Valgimigli L (2013). 3-pyridinols and 5-pyrimidinols: Tailor-made for use in synergistic radical-trapping co-antioxidant systems. Beilstein J. Org. Chem..

[56] Tundis R (2012). Antioxidant and anti-cholinesterase activity of Globularia meridionalis extracts and isolated constituents. Nat. Prod. Commun..

[57] Kayashima T, Katayama T (2002). Oxalic acid is available as a natural antioxidant in some systems. Biochim. Biophys. Acta - Gen. Subj..

[58] Ishihara Y (2011). Antioxidant properties of rare sugar D-allose: Effects on mitochondrial reactive oxygen species production in Neuro2A cells. J. Biosci. Bioeng..

[59] Maimulyanti A, Prihadi AR (2015). Chemical composition, phytochemical and antioxidant activity from extract of Etlingera elatior flower from Indonesia. J. Pharmacogn. Phytochem..

[60] Park S. Y. (2014). Ethyl linoleate from garlic attenuates lipopolysaccharide-induced pro-inflammatory cytokine production by inducing heme oxygenase-1 in RAW264.7 cells. Int. Immunopharmacol..

[61] Nishimura Y (2018). Lauric Acid Alleviates Neuroinflammatory Responses by Activated Microglia: Involvement of the GPR40-Dependent Pathway. Neurochem. Res..

[62] Servi H. (2020). Chemical composition and biological activities of endemic Tripleurospermum conoclinium. Flavour Fragr. J..

[63] Riccobono, L. *et al.* Chemical composition of volatile and fixed oils from of Salvia argentea L. (Lamiaceae) growing wild in Sicily. *Nat. Prod. Res.* **30**, 25–34 (2016). 10.1080/14786419.2015.1030742 25880372

[64] Kong Y, Jiang B, Luo X (2018). Gut microbiota influences Alzheimer’s disease pathogenesis by regulating acetate in Drosophila model. Future Microbiol..

[65] Zhang L (2017). Altered Gut Microbiota in a Mouse Model of Alzheimer’s Disease. J. Alzheimer’s Dis..

[66] Ho L (2018). Protective roles of intestinal microbiota derived short chain fatty acids in Alzheimer’s disease-type beta-amyloid neuropathological mechanisms. Expert Rev. Neurother..

[67] Youn K (2014). Oleic acid and linoleic acid from tenebrio molitor larvae inhibit BACE1 activity in vitro: Molecular docking studies. J. Med. Food.

[68] Lee E. (2013). Effect of conjugated linoleic acid, μ-calpain inhibitor, on pathogenesis of Alzheimer’s disease. Biochimica et Biophysica Acta (BBA) - Molecular and Cell Biology of Lipids.

[69] Kareti SR, Subash P (2020). In silico exploration of anti-Alzheimer’s compounds present in methanolic extract of Neolamarckia cadamba bark using GC–MS/MS. Arab. J. Chem..

[70] Amtul Z, Westaway D, Cechetto DF, Rozmahel RF (2011). Oleic acid ameliorates amyloidosis in cellular and mouse models of alzheimer’s disease. Brain Pathol..

[71] Polis, B., Gurevich, V., Assa, M. & Samson, A. O. Norvaline restores the BBB integrity in a mouse model of alzheimer’s disease. *Int. J. Mol. Sci.* **20**, (2019). 10.3390/ijms20184616 PMC6770953 31540372

[72] Lin CH (2020). Lactulose and melibiose attenuate MPTP-induced parkinson’s disease in mice by inhibition of oxidative stress, reduction of neuroinflammation and up-regulation of autophagy. Front. Aging Neurosci..

[73] Lee GC (2015). The potential of lactulose and melibiose, two novel trehalase-indigestible and autophagy-inducing disaccharides, for polyQ-mediated neurodegenerative disease treatment. Neurotoxicology.

[74] Che H (2018). A comparative study of EPA-enriched ethanolamine plasmalogen and EPA-enriched phosphatidylethanolamine on Aβ42 induced cognitive deficiency in a rat model of Alzheimer’s disease. Food Funct..

[75] Ewing, B., Hillier, L., Wendl, M. C. & Green, P. Base-Calling of Automated Sequencer Traces Using Phred . I . Accuracy Assessment. 175–185 (2005). 10.1101/gr.8.3.175 9521921

[76] Magoč T, Salzberg SL (2011). FLASH: Fast length adjustment of short reads to improve genome assemblies. Bioinformatics.

[77] Edgar RC (2010). Search and clustering orders of magnitude faster than BLAST. Bioinformatics.

[78] Edgar RC (2013). UPARSE: Highly accurate OTU sequences from microbial amplicon reads. Nat. Methods.

[79] Edgar RC, Haas BJ, Clemente JC, Quince C, Knight R (2011). UCHIME improves sensitivity and speed of chimera detection. Bioinformatics.

[80] Bernal A, Ear U, Kyrpides N (2001). Genomes OnLine Database (GOLD): A monitor of genome projects world-wide. Nucleic Acids Res..

[81] Nilsson, R. H. *et al.* Towards a unified paradigm for sequence-based identification of fungi. 5271–5277 (2013). 10.1111/mec.12481 24112409

[82] Cole J.R. (2009). The Ribosomal Database Project : improved alignments and new tools for rRNA analysis. Nucleic Acids Res
..

[83] DeSantis TZ (2006). Greengenes, a Chimera-Checked 16S rRNA Gene Database and Workbench Compatible with ARB. Appl. Environ. Microbiol..

[84] Penazzi L (2017). DMSO modulates CNS function in a preclinical Alzheimer’s disease model. Neuropharmacology.

[85] Kumar A, Darreh-Shori T (2017). DMSO: A mixed-competitive inhibitor of human acetylcholinesterase. ACS Chem. Neurosci..

[86] Krause TL, Bittner GD (1990). Rapid morphological fusion of severed myelinated axons by polyethylene glycol. Proc. Natl. Acad. Sci..

[87] Luo J, Borgens R, Shi R (2004). Polyethylene glycol improves function and reduces oxidative stress in synaptosomal preparations following spinal cord injury. J. Neurotrauma.

[88] Baptiste DC (2009). Systemic polyethylene glycol promotes neurological recovery and tissue sparing in rats after cervical spinal cord injury. J. Neuropathol. Exp. Neurol..

[89] Fiehn O. (2016). Metabolomics by gas chromatography-mass spectrometry: Combined targeted and untargeted profiling. Curr. Protoc. Mol. Biol..

[90] Cao Y, Chtarbanova S, Petersen AJ, Ganetzky B (2013). Dnr1 mutations cause neurodegeneration in Drosophila by activating the innate immune response in the brain. Proc. Natl. Acad. Sci. U. S. A..

