# Supplementary material for: Kefir metabolites in a fly model for Alzheimer’s disease
Source: Sci Rep. 2021 May 27;11:11262. doi: 10.1038/s41598-021-90749-8 (PMC8160324; doi:10.1038/s41598-021-90749-8)
Supplement: Supplementary file 1 — Supplementary Information 1. [file 41598_2021_90749_MOESM1_ESM.pdf]

Kefir metabolites in a fly model for Alzheimer's Disease

Letícia Leandro Batista[1*], Serena Mares Malta[1], Heitor Cappato Guerra Silva[1], Luiza Diniz Ferreira Borges[1], Lays Oliveira Rocha[1], Jéssica Regina da Silva[1], Tamiris Sabrina Rodrigues[1], Gabriela Venturini[2], Kallyandra Padilha[3], Alexandre da Costa Pereira[2], Foued Salmen Espindola[1], Carlos Ueira-Vieira[1*].

Excluído: Silva

[1] Institute of Biotechnology, Universidade Federal de Uberlândia, Uberlândia – Brazil.

[2] Department of Genetics, Harvard Medical School, Boston, MA, USA.

[3] Laboratory of Genetics and Molecular Cardiology, Heart Institute, University of São Paulo Medical School, São Paulo, SP, Brazil.

*Corresponding authors: batista@uni-mainz.de and ueira@ufu.br

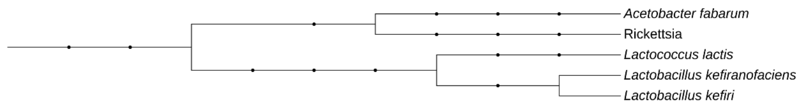

Supplementary Figure S1. Phylogenetic tree of the OTUs found in kefir microbiota.

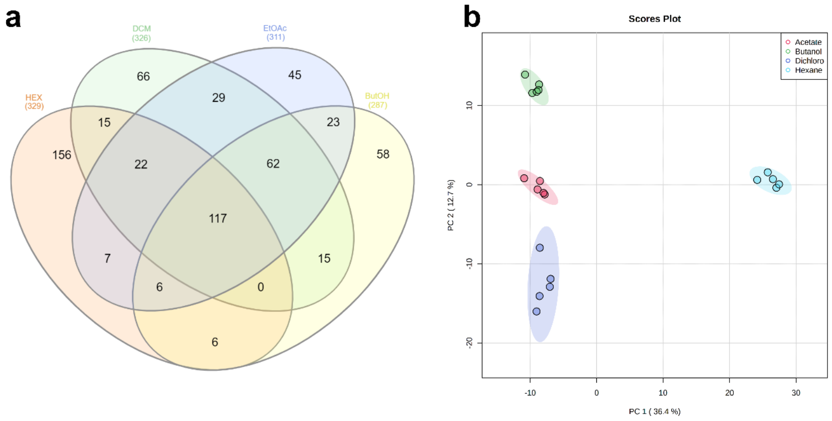

Supplementary Figure S2 a. Venn diagram of compounds present in each fraction **b**. PCA analysis of fractions metabolites (MetaboAnalyst).

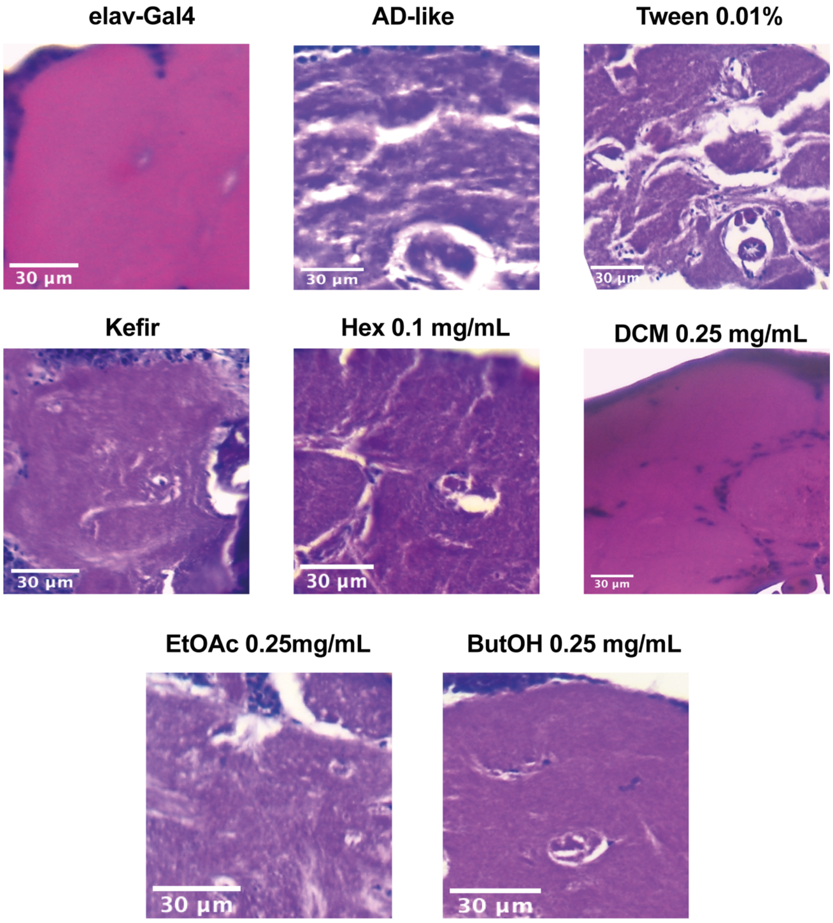

Supplementary Figure S3. Representative histopathology analysis of elav-Gal4 (control), and AD-like flies (non-treated, and treated with kefir, Tween80 0.01% or fractions). N = 10.
